# Effects of stressful life events on ART treatment outcomes: a prospective study on the role of individual and personality characteristics and psychological health conditions

**DOI:** 10.3389/fpsyg.2026.1915014

**Published:** 2026-08-31

**Authors:** Maria Clelia Zurlo, Federica Vallone, Maria Francesca Cattaneo Della Volta

**Affiliations:** 1 Dynamic Psychology Laboratory, Department of Humanities, University of Naples Federico II, Naples, Italy; 2 Department of Political Sciences, University of Naples Federico II, Naples, Italy

**Keywords:** coping strategies, infertility, psychological health, stressful life events, treatment success

## Abstract

**Background:**

Infertility research highlighted that early exposure to stressful life events (SLEs) and childhood adversities are significantly associated not only with women’s psychological health, but also with outcomes of assisted reproductive technologies. Nevertheless, there is a lack of studies adopting a multidimensional and transactional approach exploring how early life and family-related stressors potentially influence treatment outcomes through multiple interacting variables.

**Objective:**

The present study aimed to test the associations between stressful life events and ART treatment outcomes (Failure/Success) by also exploring the potential mediating role of psychological health conditions (State-Anxiety and Depression) and the moderating role of socio-demographics, infertility-related parameters, and personality characteristics.

**Methods:**

A prospective study was conducted. At the beginning of ART treatment (T1, 2020), 130 infertile women completed self-report measures assessing individual (socio-demographics) and personality (coping strategies) characteristics, fertility-related parameters, SLEs (In Family Pre-existing Pregnancy Difficulties; Problems in Family of Origin; Health Problems in Childhood), and psychological health conditions (State-Anxiety/Depression). After 5 years (T2, 2025), medical records were reviewed to verify treatment outcomes. Main/mediating/moderating hypotheses were tested by using Correlational-Analyses and Hayes-PROCESS-tool.

**Results:**

*In Family Pre-existing Pregnancy Difficulties* was found both directly and indirectly associated, partially *via* Depression, to ART treatment outcome, while the impact of *Problems in Family of Origin* and *Health Problems in Childhood* was fully mediated by Depression. Avoidant coping strategies were found to be a significant moderator exacerbating the negative impact of *In Family Pre-existing Pregnancy Difficulties* on Depression. Conversely, higher educational levels, along with the adoption of positive attitude and social support coping strategies, played significant buffering moderation roles, acting as pivotal resources.

**Conclusion:**

These findings underscore the influence of stressful life events on ART outcomes *via* women’s depressive symptomatology and the role of specific moderating variables that should be addressed through routine psychological screening and multidisciplinary counselling interventions in ART centers to enhance both mental health and treatment success for infertile women.

## 1 Introduction

A substantial body of infertility research has documented the detrimental effects of current perceived infertility-related stressors on both women’s psychological health conditions (Domínguez-Salas et al., 2026; Gameiro et al., 2012; Zurlo et al., 2017) and treatment outcomes (Boivin et al., 2011; Öztürk et al., 2021; Purewal et al., 2018; Zurlo et al., 2025). Early research primarily focused on the impact of current infertility-related stressors and treatment burden, while further research has increasingly highlighted that also stressful experiences occurring earlier in life, such as adverse childhood experiences and Stressful Life Events (SLEs), may exert significant effects on infertile women’s psychological health conditions as well as on treatment outcome. Specifically, research in this field has demonstrated that SLEs exert long-term and pervasive effects on psychological health conditions (Abate et al., 2025; Dalechek et al., 2024; Xu et al., 2024), on vulnerability to psychopathology (McEwen, 1998), and on perceived physical health (Hughes et al., 2017; Petruccelli et al., 2019).

Considering physical health, adverse childhood experiences have been consistently associated with a higher risk of cardiovascular disease, type 2 diabetes, chronic obstructive pulmonary disease, autoimmune disorders, chronic pain syndromes, and increased multimorbidity in adulthood (Felitti et al., 1998; Hughes et al., 2017). These associations are thought to be mediated by long-term alterations in neuroendocrine regulation, inflammatory pathways, and health-risk behaviors (Danese and McEwen, 2012).

With specific reference to reproductive health, research highlighted that SLEs have a significant influence on female reproductive functions and reproductive trajectories, and are linked to menstrual irregularities, infertility, pregnancy complications, perinatal depression, and adverse neonatal outcomes (Harville et al., 2010; Kerkar et al., 2021; Smith et al., 2015). From this perspective, exposure to childhood adversities – including abuse, neglect, parental loss, and family dysfunction – has been consistently linked to alterations in pubertal timing, menstrual regulation, ovulatory function, luteal phase abnormalities, and impaired ovarian function (Belsky et al., 2015; Ge et al., 2001; Jacobs et al., 2015; Yuan et al., 2026). This is also in terms of accelerated reproductive aging, indicated by reduced ovarian reserve markers and earlier menopause (Kling et al., 2023; Ryan et al., 2021). However, SLEs have also been linked to adverse reproductive outcomes, including lower pregnancy likelihood, miscarriage, and obstetric complications such as preterm birth and low birth weight (Christiaens et al., 2015; Kerkar et al., 2021; Mamun et al., 2023; Mersky and Lee, 2019; Swift et al., 2024).

Altogether, these data are confirmed within infertility research, which highlighted that infertile individuals report a higher prevalence of stressful life events than fertile controls, and, particularly events related to family of origin, childhood health, or prior pregnancy difficulties, thus suggesting enduring effects of early developmental stress (Wischmann, 1998; Wischmann et al., 2009; Geisler et al., 2020; Gleason et al., 2020; Zurlo et al., 2019).

Although there is evidence linking SLEs to assisted reproductive technology outcomes, the findings are still mixed. Indeed, some studies reported no significant impact on pregnancy outcomes (Pottinger et al., 2016), whereas others highlighted that negative life events prior to treatment are associated with lower pregnancy rates and higher likelihood of treatment failure (Ebbesen et al., 2009; Yilmaz et al., 2015).

Moreover, a multidimensional approach to the issue remains limited, and, in particular, few studies have simultaneously explored the combined effects of SLEs while considering variables that may act as mediators or moderators of their effects.

In this perspective, empirical research on coping strategies and their association with infertility treatment and psychological outcomes has produced heterogeneous results, reflecting the situational nature of coping. Zurlo et al. (2025) demonstrated that Problem Solving and Positive Attitude significantly act as protective strategies, buffering the negative impact of infertility-related stress on treatment outcomes, whereas avoidance-oriented coping is linked to poorer outcomes. Coping strategies also moderate the association between previous stressful life events and psychological well-being, with Positive Attitude, Problem Solving, and Social Support mitigating the negative impact on quality of life among infertile women (Zurlo et al., 2019). Similarly, Hudson et al. (2025) found that coping strategies – such as positive cognitive restructuring, problem-focused coping, and support seeking – attenuate the association between stressful life events and symptoms of anxiety and depression in young people, whereas avoidance coping amplifies psychological distress.

Complementing this perspective, research has highlighted the relevance of exploring and deepening the complex process (direct and indirect effects) linking – *over time* – life events to psychological health conditions, and their joint associations with mental and physical diseases (Roos et al., 2020). From this perspective, the longitudinal association between adverse experiences, psychological health conditions, and physical symptoms has been well-established in the literature, and increasing evidence supports the mediating role of psychological health conditions, mainly anxiety and depression, in linking early adversities and stressful events with health-related outcomes (Hammond et al., 2019; Lee et al., 2022; Li et al., 2024; McCall-Hosenfeld et al., 2014). Specifically, research conducted by Hammond et al. (2019) and by Lee et al. (2022) showed that anxiety and depression mediated the longitudinal association between adverse childhood experiences and subsequent somatic symptoms in adolescence. In the same direction, McCall-Hosenfeld et al. (2014) provided evidence on the mediating role of depression linking trauma exposure and somatic symptom severity in patients with chronic pain. Similarly – and more recently – Li et al. (2024) found that depressive symptoms partially mediated the relationship between adverse childhood experiences and chronic health conditions in adulthood.

Overall, these findings support the hypothesis of adopting a transactional approach, in which previous stressful life events reported by infertile women could be considered as exerting an influence on ART treatment outcomes, and, in turn, such effects could be mediated by their current psychological health conditions and moderated by personality characteristics such as coping strategies adopted to face infertility and infertility treatment.

### 1.1 The present study

Drawing on the literature described above, the present prospective study enrolled infertile women at the beginning of medical treatment (T1) to examine psychological predictors, as well as mediating and moderating variables, associated with ART treatment outcomes at the 5-year follow-up (T2). On this basis, the following hypotheses were specified and empirically tested:

*Hypothesis One* (H1) – *Main effects*. Stressful Life Events assessed at T1 (In Family Pre-existing Pregnancy Difficulties, Problems in Family of Origin, and Health Problems in Childhood) will be significantly associated with a lower likelihood of Treatment Success.

*Hypothesis Two* (H2) – *Mediating Effects*. Psychological Health conditions (State-Anxiety and Depression) will play as mediators in the association between Stressful Life Events and Treatment Outcome (Success/Failure).

*Hypothesis Three* (H3) – *Moderating Effects*. Socio-demographic characteristics, infertility-related parameters, and adopted Coping Strategies will significantly moderate/buffer the relationship of Stressful Life Events with Psychological Health conditions and Treatment Success.

## 2 Materials and methods

### 2.1 Sampling and procedure

The present study used a prospective design targeting women who referred to centers of reproductive medicine in Italy to undergo ART treatments in 2020 (T1 in the present study). Specifically, in the first implementation phase, chairpersons of four centers, highly specialized in reproductive health, were asked to endorse the project and provide authorization to approach their patients. To be included in the study, women had to meet the following inclusion criteria: (a) having received a diagnosis of infertility; (b) undergoing the first ART treatment; (c) being able to read and understand written Italian. At the time of data collection, 150 women were considered eligible and directly approached by a trained psychologist, who invited them to participate. Women received all the information about the project aims and their rights to participate (to not participate or to withdraw from the survey at any time, without giving any explanation). They were also assured about the confidentiality of their data and informed that their responses would be used exclusively for research purposes. Participation in the project was clarified to be on a voluntary basis. The study was approved by the Ethical Committee of Psychological Research of the University of Naples Federico II (IRB: 34/2019) and conducted in accordance with the 1964 Helsinki Declaration and its later amendments. All precautions were taken to protect participants’ privacy and confidentiality, and informed consent was obtained from each woman prior to inclusion.

Overall, 130 women out of 150 agreed to participate (*Response Rate* = 86.7%). An *a priori* power analysis was conducted for multiple linear regression models by using G*Power (Version 3.1.9.7). The results indicated that a sample size of 107 participants – for mediation analysis – and a sample size of 92 – for moderation analysis – would be required in order to reach a 0.85 power level, with a medium effect size (*f^2^* = 0.10; *α* = 0.05). Furthermore, for specifically testing indirect effects of mediation analyses, a power analysis simulation was carried out by using the Monte Carlo application (sample size of *N* = 130; 5,000 replications; medium effect size; *α* = 0.05; Schoemann et al., 2017). The results further confirmed that the sample size of 130 women was large enough to provide satisfactory power for mediation analyses (indirect effects statistical power = 0.85) and to decrease estimation error for all the analyses carried out.

Participants were asked to complete a written survey lasting 25–30 min in a quiet room within the center. One of the authors of the present study was always present during the completion of the questionnaire to address any questions raised by women. The surveys were completed in all the sections; therefore, there were no missing data to handle. All participants completing the survey were in a heterosexual relationship and had been diagnosed with primary infertility.

**Figure 1:**
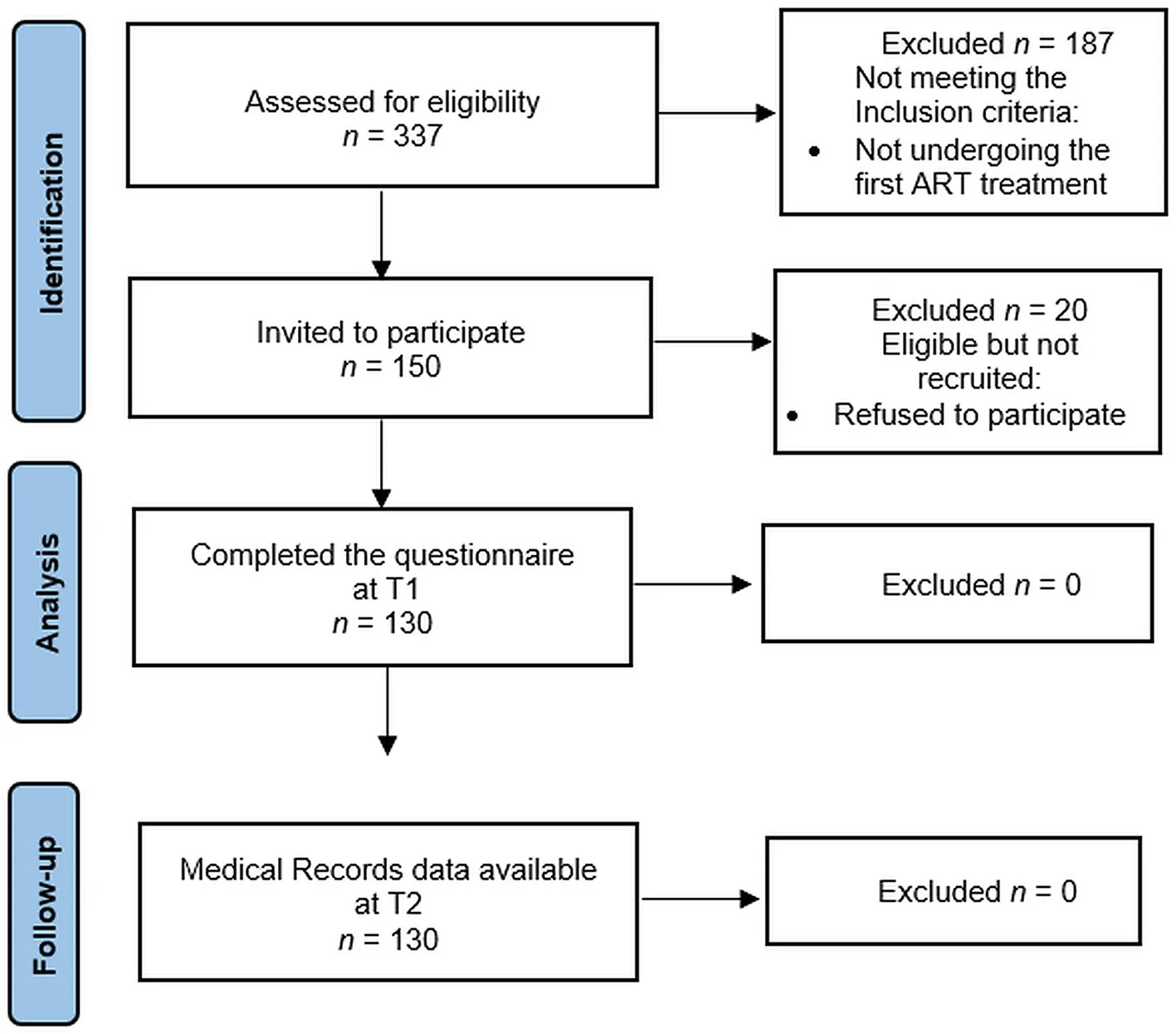
STROBE flowchart: study participants.

In line with the prospective study design, in 2025 (T2 in the present study), center chairpersons were contacted and asked to provide data on women’s treatment outcomes (Failure/Success) along with information on length of infertility and the number of medical cycles undergone. None of the women enrolled at T1 and included in the present study had changed the center. Therefore, complete follow-up data were obtained. The STrengthening the Reporting of OBservational studies in Epidemiology (STROBE) flowchart illustrating participants’ recruiting process and study participants is presented in Figure 1.

### 2.2 Measures

In 2020 (at T1), women were asked to fill out a tailored survey consisting of five sections, namely:

(1) Sociodemographic and Infertility-related Characteristics: This section assessed women’s *Age* (in years); *Educational Level* (Junior/Middle School; Senior School; College); and *Employment status* (Unemployed/Employed). A single item assessing the *Type of Diagnosis* (Male Factor, Female Factor, Combined Factor, and Unexplained diagnosis) was also included;
(2) Stressful Life Events (SLEs): the presence of stressful events was assessed by using a tailored tool specifically developed to be used in infertility research, namely the *Stress-inducing Events in the Couples’ Lives Questionnaire* (FLS; Wischmann, 1998). Women were asked to respond to three single items related to stressful events in their family of origin and in own childhood. Specifically, women were asked if they had experienced *Stressful events in the family of origin*, *In Family Pre-existing pregnancy difficulties,* and *Health problems in childhood* (No = Absence/ Yes = Presence).
(3) Coping strategies: coping strategies adopted by the women to deal with infertility were assessed by using the *Coping Orientations to Problem Experienced-New Italian Version* (COPE-NVI; Sica et al., 2008). The COPE-NVI consists of 60 items on a 4-point Likert scale ranging from one (“I usually do not do this at all”) to four (“I usually do this a lot”) divided into five subscales: *Problem Solving* (12 items; Cronbach’s *α* = 0.83), *Social Support* (12 items; Cronbach’s *α* = 0.88), *Avoiding* (16 items; Cronbach’s *α* = 0.70), *Positive Attitude* (12 items; Cronbach’s *α* = 0.76), and *Turning to Religion* (8 items; Cronbach’s *α* = 0.85). Coping Strategies scores were also converted into percentages based on the cut-off points reported in the Italian validation study (Sica et al., 2008; *Problem Solving* Cut-off = 32.0; *Social Support* Cut off = 27.7; *Avoiding* Cut off = 23.5; *Positive Attitude* Cut off = 30.9; *Turning to Religion* Cut off = 22.7) in order to better identify and clearly display women who highly adopt a certain coping strategy – thus to a greater extent than the normative reference group.
(4) State-Anxiety: women’s perceived levels of state-anxiety were assessed by using the State scale from the State–Trait Anxiety Inventory (STAI-Y; Pedrabissi and Santinello, 1989; Spielberger, 1972). It consists of 20 items on a 4-point Likert scale ranging from one (“Not at all”) to four (“Very much”). The total score ranges from 20 to 80 (Cronbach’s *α* = 0.91). In order to identify women reporting clinically relevant levels of State-Anxiety, scores were also converted into percentages by using the clinical cut-off points provided in the Italian validation study (Pedrabissi and Santinello, 1989; *State-Anxiety* Cut off = 39.9).
(5) Depression: women’s perceived levels of depression were assessed by using the Edinburgh Depression Scale (EDS; Benvenuti et al., 1999; Murray and Cox, 1990). It consists of 10 items on a 4-point Likert scale ranging from zero (“Not at all”) to three (“Most of the time”). The total score ranges from 0 to 30 (Cronbach’s *α* = 0.78; Cut off = 9). In order to identify women reporting clinically relevant levels of Depression, scores were also converted into percentages by using the clinical cut-off points provided in the Italian validation study (Benvenuti et al., 1999; *Depression* Cut off = 9).

After 5 years (T2, 2025), medical records were used to collect data on the ART treatment outcomes (Failure or Success). Live birth – rather than the positive pregnancy test – was considered the criterion for treatment success. Information on duration of infertility (in years) and treatment cycles (number) was also registered at T2.

### 2.3 Data analysis

Preliminary to data analysis, women were prospectively classified into two groups according to the treatment outcome registered at T2. The two groups were labelled as “Treatment Success” and “Treatment Failure,” respectively. Therefore, descriptive analyses were computed for all the study variables, and scores were also compared according to the group membership. Specifically, Student’s *t*-test was used for continuous variables, while *χ*^2^-Analysis was used for categorical variables. Considering the multiple comparisons, the Bonferroni–Holm correction method was used to adjust the *p*-values (*α* = 0.05), thus minimizing the risk of Type I error.

Moreover, preliminary to hypothesis testing, Spearman’s rank-order correlations were computed among the study variables. This was done to ensure the feasibility of testing the study hypotheses. Therefore, in order to test *Hypothesis One* (H1; *Main Effects*), exploring the potential main effects of each SLE on Treatment Outcomes, Logistic Regression Analyses were run (method: enter, first indicator contrast; entry criterion: *p* < 0.05; removal criterion: *p* > 0.01, and Hosmer and Lemeshow Goodness-of-fit statistic fixed at *p* > 0.05).

Afterwards, in order to test *Hypothesis Two* (H2; *Mediating Effects*), exploring the potential mediating role of Psychological Health conditions (State-Anxiety and Depression) in the associations between SLEs and Treatment Outcomes, Model 4 of Hayes’ PROCESS macro for SPSS was used (Hayes, 2017; Preacher and Hayes, 2008). Direct and indirect (mediation) analyses, bias-corrected bootstrapped test with 5,000 replications to ensure the 95% Confidence Interval (with lower and upper bounds not containing zero) were used to verify the significance of the effects (Hayes, 2017). Moreover, in order to test the models’ performance (Hosmer et al., 2013), the analyses were replicated by using Multivariable Logistic Regression Models and the discriminant ability of the models was evaluated by analyzing the Receiver Operating Characteristic (ROC) curves and the areas under the curve (AUC ≥ 0.70 indicating fair/moderate accuracy; Bujang, 2026), while calibration was explored by using the Hosmer–Lemeshow goodness-of-fit test, with a non-significant result (*p* > 0.05) indicating a good calibration.

Finally, in order to test *Hypothesis Three* (H3; *Moderating Effects*), exploring the potential moderating role of Socio-demographic and Infertility-related Characteristics and of Coping Strategies – respectively – in the associations between SLEs, Psychological Health, and Treatment Outcomes, Model 1 of Hayes’ PROCESS macro for SPSS was used (Hayes, 2017; Preacher and Hayes, 2008). The statistical significance of interaction effects was examined (*p* < 0.05). Moreover, in order to establish whether the inclusion of the interaction terms significantly improved the models, delta R-sq values (Δ*R*^2^) were reported for continuous outcomes, while *χ*^2^ values were reported for dichotomous outcomes. Simple slopes analyses were also graphically displayed. The Statistical Package for the Social Sciences (SPSS; Version 30) was employed for all analyses.

## 3 Results

### 3.1 Preliminary results

Firstly, by prospectively classifying sampled women according to the treatment outcome registered at T2, data revealed that 61 women out of 130 (46.9%) achieved a live birth and were therefore assigned to the “Treatment Success” group, while 69 women (53.1%) were included in the “Treatment Failure” group.

Table 1 shows data on Socio-demographic and Infertility-related characteristics of sampled women (Total Sample) along with comparisons drawn according to the Treatment Outcome. With respect to Socio-demographic characteristics, the data revealed statistically significant differences between the *Treatment Failure* and *Treatment Success* groups only in terms of Educational Level, with the latter displaying a higher educational background. Considering Infertility-related characteristics, findings revealed no statistically significant differences between the two groups in terms of the prevalence of different types of diagnoses yet confirmed the expected findings of a longer duration of infertility and a higher number of treatment cycles among women in the *Treatment Failure* group. However, the data also indicated that women in the *Treatment Success* group achieved pregnancy within 3 years of diagnosis, undergoing up to a maximum of 5 treatment cycles (Table 1).

**Table 1:** Socio-demographic and infertility-related characteristics of infertile women (*N* = 130) by treatment outcome at T2.

Variables	Total sample	Treatment outcome collected at T2		*p-*value	Adjusted *p*-value^a^
	*N* = 130	Treatment success *n* = 61 (46.9%)	Treatment failure *n* = 69 (53.1%)		
Variables collected at T1					
Age in years [mean ± SD]	34.2 ± 3.4	34.5 ± 3.4	33.9 ± 3.3	0.718	1.000
Employment status [*n* (%)]					
Unemployed	24 (18.5%)	12 (19.7%)	12 (17.4%)	0.456	1.000
Employed	106 (81.5%)	49 (80.3%)	57 (82.6%)		
Educational level [*n* (%)]					
Junior middle school education	18 (13.8%)	1 (1.6%)	17 (24.6%)	0.000***	0.000***
Senior school education	69 (53.1%)	35 (57.4%)	34 (49.3%)		
College education	43 (33.1%)	25 (41.0%)	18 (26.1%)		
Type of diagnosis [*n* (%)]					
Female factor	53 (40.8%)	27 (44.3%)	26 (37.7%)	0.112	0.232
Male factor	41 (31.5%)	21 (34.4%)	20 (29.0%)		
Combined factor	17 (13.1%)	9 (14.7%)	8 (11.6%)		
Unexplained	19 (14.6%)	4 (6.6%)	15 (21.7%)		
Variables collected at T2					
Duration of infertility in years [mean ± SD]	3.9 ± 1.0	2.9 ± 0.3	4.7 ± 0.8	0.000***	0.000***
Number of treatment cycles [*n* (%)]					
<3 cycles	44 (33.8%)	30 (49.2%)	14 (20.3%)	0.000***	0.000***
3–5 cycles	38 (29.2%)	31 (50.8%)	7 (10.1%)		
>5 cycles	48 (37.0%)	0 (0.0%)	48 (69.6%)		

Differences were calculated using Student’s *t*-test (mean ± standard deviations) and *χ*^2^-test [*N* (%)].

Statistically significant results are reported in bold.

*p < 0.05; **p < 0.01; ***p < 0.001.

a Adjusted *p*-values were calculated using the Holm–Bonferroni correction method.

Descriptive data and comparisons by Treatment Outcome on the presence of Stressful Life Events, the adoption of Coping Strategies, and Psychological Health Conditions are illustrated in Table 2. Specifically, findings revealed that sampled women belonging to the *Treatment Failure* group reported a statistically higher frequency of In Family Pre-existing Pregnancy Difficulties (SLE), adopted coping strategies centered on Positive Attitude to a lesser extent, while they used passive coping strategies (Avoiding and Turning to Religion coping strategies) to a greater extent than women belonging to the *Treatment Success* group. They also reported statistically significantly higher levels of Depression (Table 2).

**Table 2:** Number and percentages of women reporting absence/presence of stressful life events, low and high recourse to coping strategies, and low and high (clinically relevant) levels of state-anxiety and depression at T1 by treatment outcome at T2.

Variables collected at T1	Total sample	Treatment outcome collected at T2				*χ* ^2^ ^a^	*p* value	Adjusted *p* value^a^
	*N* = 130	Treatment Success *n* = 61		Treatment Failure *n* = 69				
	*N* (%)	*N*	(%)	*N*	(%)			
Stressful life events								
Problems in family of origin								
Absence	70 (53.9)	38	(62.3)	32	(46.4)			
Presence	60 (46.1)	23	(37.7)	37	(53.6)	3.301	0.800	0.855
In family pre-existing pregnancy difficulties								
Absence	87 (66.9)	49	(80.3)	38	(55.1)			
Presence	43 (33.1)	12	(19.7)	31	(44.9)	**9.329**	0.003**	0.021*
Health problems in childhood								
Absence	77 (59.2)	40	(65.6)	37	(53.6)			
Presence	53 (40.8)	21	(34.4)	32	(46.4)	1.915	0.211	0.844
Coping strategies								
Social support								
Low	125 (96.1)	60	(98.7)	65	(94.2)			
High	5 (3.9)	1	(1.6)	4	(5.8)	1.513	0.370	0.855
Problem solving								
Low	114 (87.7)	56	(91.8)	58	(84.1)			
High	16 (12.3)	5	(8.2)	11	(15.9)	1.800	0.285	0.855
Positive Attitude								
Low	97 (74.6)	38	(62.3)	59	(85.5)			
High	33 (25.4)	23	(37.7)	10	(14.5)	**9.210**	0.004**	0.024*
Turning to Religion								
Low	80 (61.5)	56	(91.8)	24	(34.8)			
High	50 (38.5)	5	(8.2)	45	(65.2)	**44.476**	0.000***	0.000***
Avoiding								
Low	67 (51.5)	46	(75.4)	21	(30.4)			
High	63 (48.5)	15	(24.6)	48	(69.6)	**26.221**	0.000***	0.000***
Psychological health conditions								
State-anxiety								
Low	92 (70.8)	50	(82.0)	42	(60.9)			
High	38 (29.2)	11	(18.0)	27	(39.1)	**6.967**	0.012*	0.060
Depression								
Low	101 (77.7)	57	(93.4)	44	(63.8)			
High	29 (22.3)	4	(6.6)	25	(36.2)	**16.450**	0.000***	0.000***

Differences were calculated using *χ*^2^-test [*N* (%)]. Statistically significant results are reported in bold.

*p < 0.05; **p < 0.01; ***p < 0.001.

a Adjusted *p*-values were calculated using the Holm–Bonferroni correction method.

Table 3 shows findings from Spearman’s rank-order correlations. Preliminary correlational data partially endorsed the testing of H1, as they suggested that In Family Pre-existing Pregnancy Difficulties could be the only SLE directly associated with Treatment Outcome (H1). However, the statistically significant positive associations of all three SLEs with Depression – which, in turn, was found to be significantly negatively related to Treatment Success – fully endorsed the testing of mediating hypotheses (H2). Furthermore, the statistically significant inter-correlations among all the study variables provided adequate evidence supporting the testing of moderating hypotheses (H3).

**Table 3:** Intercorrelations among study variables (*N* = 130).

Variables	1	2	3	4	5	6	7	8	9	10	11	12	13	14	15	16	17	18	19	20
Sociodemographic characteristics																				
1. Age	1																			
2. Employment Status	0.20^*^	1																		
3. Educational Level	0.19^*^	−0.04	1																	
Infertility-related parameters																				
4. Female Diagnosis	−0.12	−0.05	0.06	1																
5. Male Diagnosis	0.04	−0.02	0.18*	−0.56^**^	1															
6. Combined Diagnosis	−0.06	0.07	−0.01	−0.32^**^	−0.26^**^	1														
7. Unexplained Diagnosis	0.17	0.03	−0.30^**^	−0.34^**^	−0.28^**^	−0.16	1													
8. Duration of Infertility	0.25**	0.16	−0.15^*^	−0.17^*^	0.09	−0.08	0.20^*^	1												
9. Number of Treatment Cycles	−0.13	−0.01	−0.40^**^	−0.06	−0.07	0.02	0.16	0.50^**^	1											
Stressful life events																				
10. Problems in Family of Origin	0.03	−0.08	−0.04	−0.14	−0.06	0.05	0.23^**^	0.08	0.27^**^	1										
11. In Family Pre-existing Pregnancy Difficulties	0.00	−0.17	−0.08	−0.15	0.01	0.11	0.08	0.30^**^	0.31^**^	0.14	1									
12. Health Problems in Childhood	0.02	0.07	−0.02	−0.02	−0.13	0.00	0.19^*^	−0.09	−0.08	0.17^*^	−0.02	1								
Coping strategies																				
13. Social Support	0.03	0.13	0.33^**^	−0.06	0.09	0.09	−0.15	−0.27^**^	−0.60^**^	−0.37^**^	−0.01	0.00	1							
14. Problem Solving	0.16	0.07	0.29^**^	−0.07	0.05	0.05	−0.07	−0.10	−0.45^**^	0.10	0.06	0.02	0.43^**^	1						
15. Positive Attitude	0.20^*^	0.02	0.19^*^	−0.03	0.08	0.08	−0.07	−0.12	−0.30^**^	0.00	−0.22^*^	−0.02	0.10	0.04	1					
16. Turning to Religion	−0.31^**^	−0.05	−0.38^**^	−0.08	−0.10	−0.02	0.27^**^	0.22^**^	0.65^**^	0.25^**^	0.20^*^	0.11	−0.50^**^	−0.52^**^	−0.24^**^	1				
17. Avoiding	−0.20^*^	0.10	−0.53^**^	−0.05	0.05	−0.22^*^	0.22^*^	0.32^**^	0.73^**^	0.19^*^	0.08	−0.10	−0.60^**^	−0.39^**^	−0.23^**^	0.59^**^	1			
Psychological health conditions																				
18. State-Anxiety	−0.02	0.15	−0.05	−0.06	0.07	−0.01	−0.01	0.02	0.16	0.07	−0.07	−0.03	−0.22^*^	−0.08	−0.04	0.11	0.30^**^	1		
19. Depression	−0.08	0.01	−0.37^**^	−0.13	−0.05	0.00	0.25^**^	0.24^**^	0.43^**^	0.25^**^	0.23^*^	0.21^*^	−0.45^**^	−0.09	−0.26^**^	0.38^**^	0.38^**^	0.21^*^	1	
Treatment outcome																				
20. Treatment Success	0.09	−0.03	0.27^**^	0.07	0.06	0.05	−0.21^*^	−0.53^**^	−0.58^**^	−0.16	−0.27^**^	−0.12	0.45^**^	0.05	0.29^**^	−0.51^**^	−0.47^**^	−0.24^**^	−0.46^**^	1

Spearman’s correlations values. **p < 0.01; *p < 0.05.

### 3.2 Hypothesis one (H1) – main effects

With respect to H1, and in line with the correlation analysis, findings from Logistic Regression Analyses revealed that the presence of *In Family Pre-existing Pregnancy Difficulties* was the only Stressful Life Event (SLE) statistically significantly associated with a lower likelihood of belonging to the Treatment Success group (O.R. = 0.300; 95% C.I. = 0.136–0.611; *p* = 0.003). Indeed, no statistically significant associations were found either for *Problems in the Family of Origin* (O.R. = 0.523; 95% C.I. = 0.260–1.056; *p* = 0.071) or *Health Problems in Childhood* (O.R. = 0.607; 95% C.I. = 0.299–1.234; *p* = 0.168). Therefore, H1 was fully supported for *In Family Pre-existing Pregnancy Difficulties* yet not for the other two SLEs.

### 3.3 Hypothesis two (H2) – mediating effects

With respect to H2, findings fully supported the mediating role of Depression in the associations between all Stressful Life Events (SLEs) and Treatment Outcome (Supplementary Table S1). Furthermore, all these models – replicated by using Multivariable Logistic Regression Models – revealed adequate discriminant ability (ROC curves illustrated in Supplementary Figure S1; AUC > 0.70) and calibration (Hosmer–Lemeshow goodness-of-fit test with *p* > 0.05).

**Figure 2:**
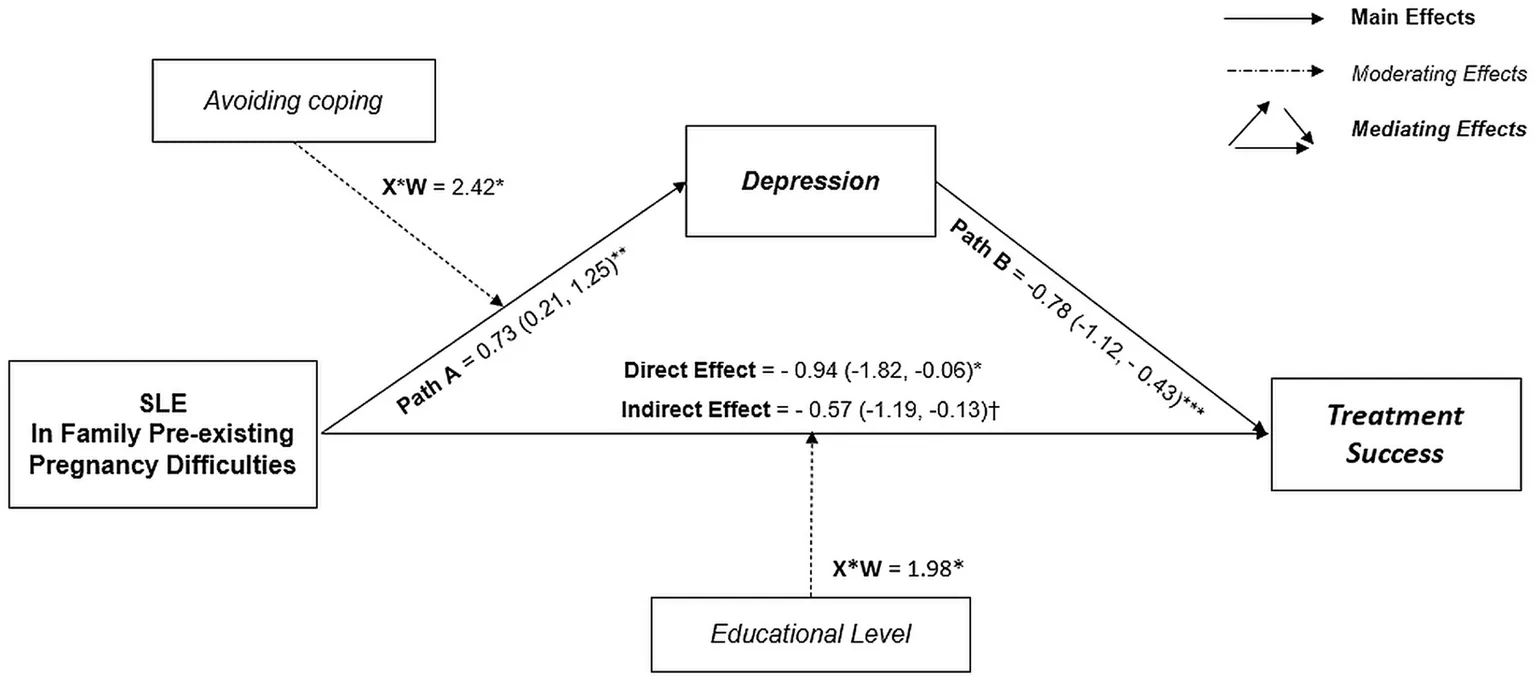
Summary: Statistically significant mediating and moderating effects on the associations between SLE-in family pre-existing pregnancy difficulties and ART treatment outcome (success). SLE, stressful life events; ^†^Partial mediation; Path A = effect of independent variable on mediator, Path B = effect of mediator on dependent variable, Direct effect = effect of independent variable on dependent variable controlling for the mediator, Indirect effect = effect of independent variable on dependent variable through the mediator; X*W = interaction effect between independent variable (X) and moderating variable (W) on dependent variable; Only significant effects were displayed *p < 0.05. **p < 0.01. ***p < 0.001.

Specifically, the association between *In Family Pre-existing Pregnancy Difficulties* and *Treatment Success* (Figure 2) was partially mediated by Depression (Nagelkerke *R*^2^ = 0.320, *p* < 0.001), as the confidence interval for its indirect effect did not contain zero (Effect = − 0.57, C.I. = − 1.19 to −0.13). With respect to model performance, the AUC was 0.782 (C.I. = 0.706–0.859) and the Hosmer–Lemeshow goodness-of-fit test was non-significant (*χ*^2^ = 5.609, *df* = 6, *p* = 0.468), revealing adequate discriminant ability and calibration.

**Figure 3:**
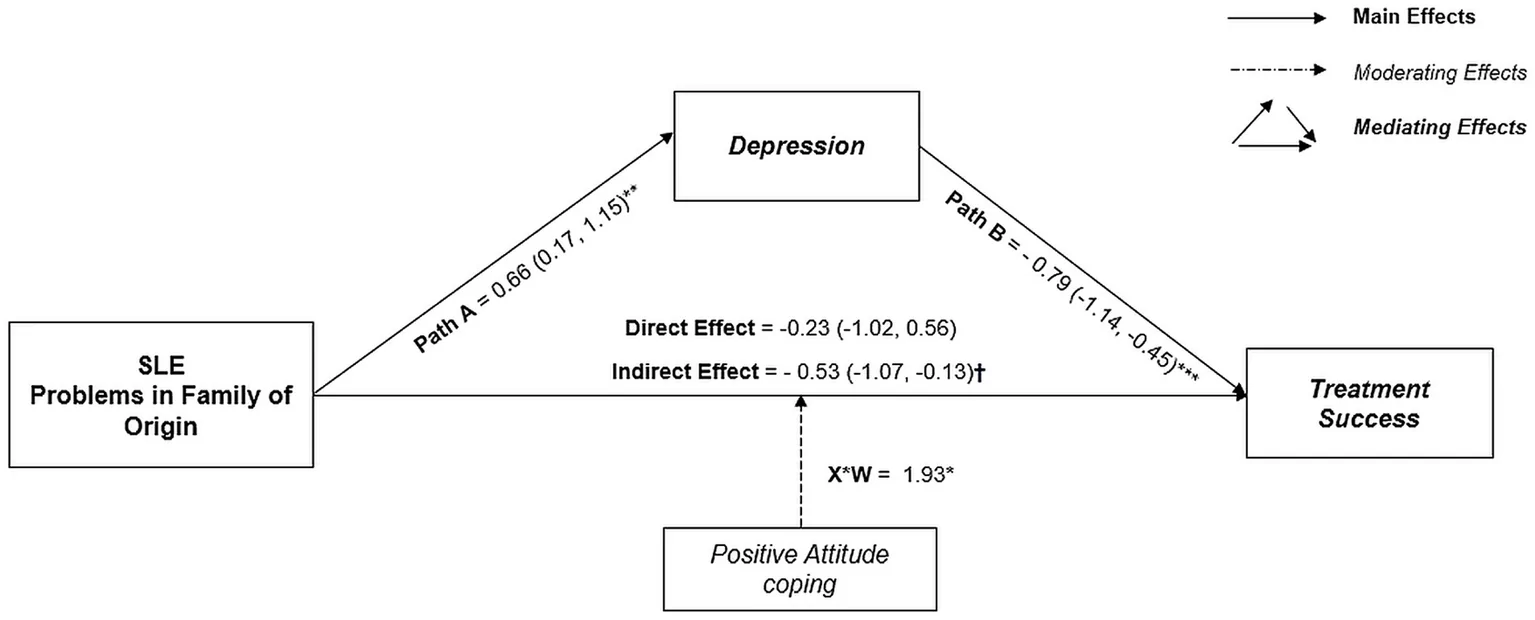
Summary: Statistically significant mediating and moderating effects on the associations between SLE-problem in family of origin and ART treatment outcome (success). SLE, stressful life events; ^†^full mediation; path *A* = effect of independent variable on mediator; path *B* = effect of mediator on dependent variable; direct effect = effect of independent variable on dependent variable controlling for the mediator; indirect effect = effect of independent variable on dependent variable through the mediator; *X* * *W* = i*nteraction effect between independent variable* (*X*) and moderating variable (*W*) on dependent variable. Only significant effects were displayed: *p < 0.05 **p < 0.01 ***p < 0.001.

**Figure 4:**
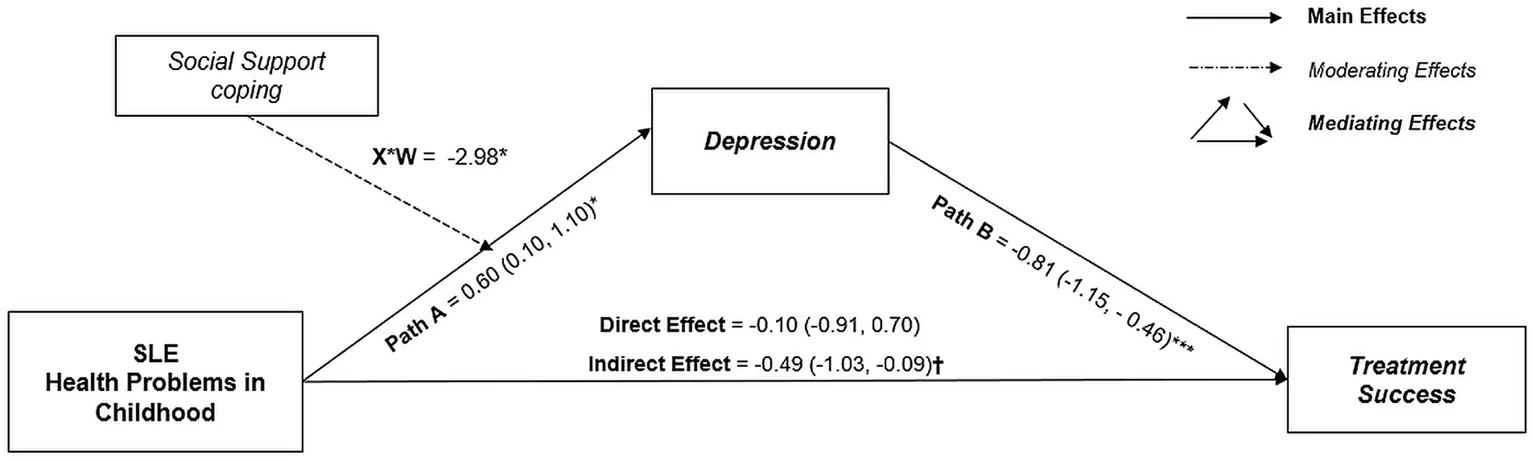
Summary: Statistically significant mediating and moderating effects on the associations between SLE-health problems in childhood and ART treatment outcome (success). SLE, stressful life events; ^†^full mediation; Path A = effect of independent variable on mediator, Path B = effect of mediator on dependent variable, Direct effect = effect of independent variable on dependent variable controlling for the mediator, Indirect effect = effect of independent variable on dependent variable through the mediator; X*W = interaction effect between independent variable (*X*) and moderating variable (*W*) on dependent variable; only significant effects were displayed. *p < 0.05. **p < 0.01. ***p < 0.001.

Moreover, given the non-significance of the Direct Effects, full mediation effects of Depression were found for the associations of both *Problems in the Family of Origin* (Figure 3; Nagelkerke *R*^2^ = 0.286, *p* < 0.001; Effect = −0.53, C.I. = −1.07 to −0.13) and *Health Problems in Childhood* (Figure 4; Nagelkerke *R*^2^ = 0.284, *p* < 0.001; Effect = −0.49, C.I. = − 1.03 to −0.09) with Treatment Success. Adequate discriminant ability and calibration was also supported for these predictive models (*Problems in the Family of Origin* and *Depression* on Treatment Outcome: AUC = 0.763, C.I. = 0.683–0.844; *χ*^2^ = 3.334, *df* = 6, *p* = 0.766; *Health Problems in Childhood* and *Depression* on Treatment Outcome: AUC = 0.746, C.I. = 0.683–0.845; *χ*^2^ = 6.017, *df* = 6, *p* = 0.421).

Finally, as expected from correlation analyses, no evidence supported the mediating role of state-anxiety. Therefore, H2 was fully supported for Depression yet not for state-anxiety.

### 3.4 Hypothesis three (H3) – moderating effects

**Figure 5:**
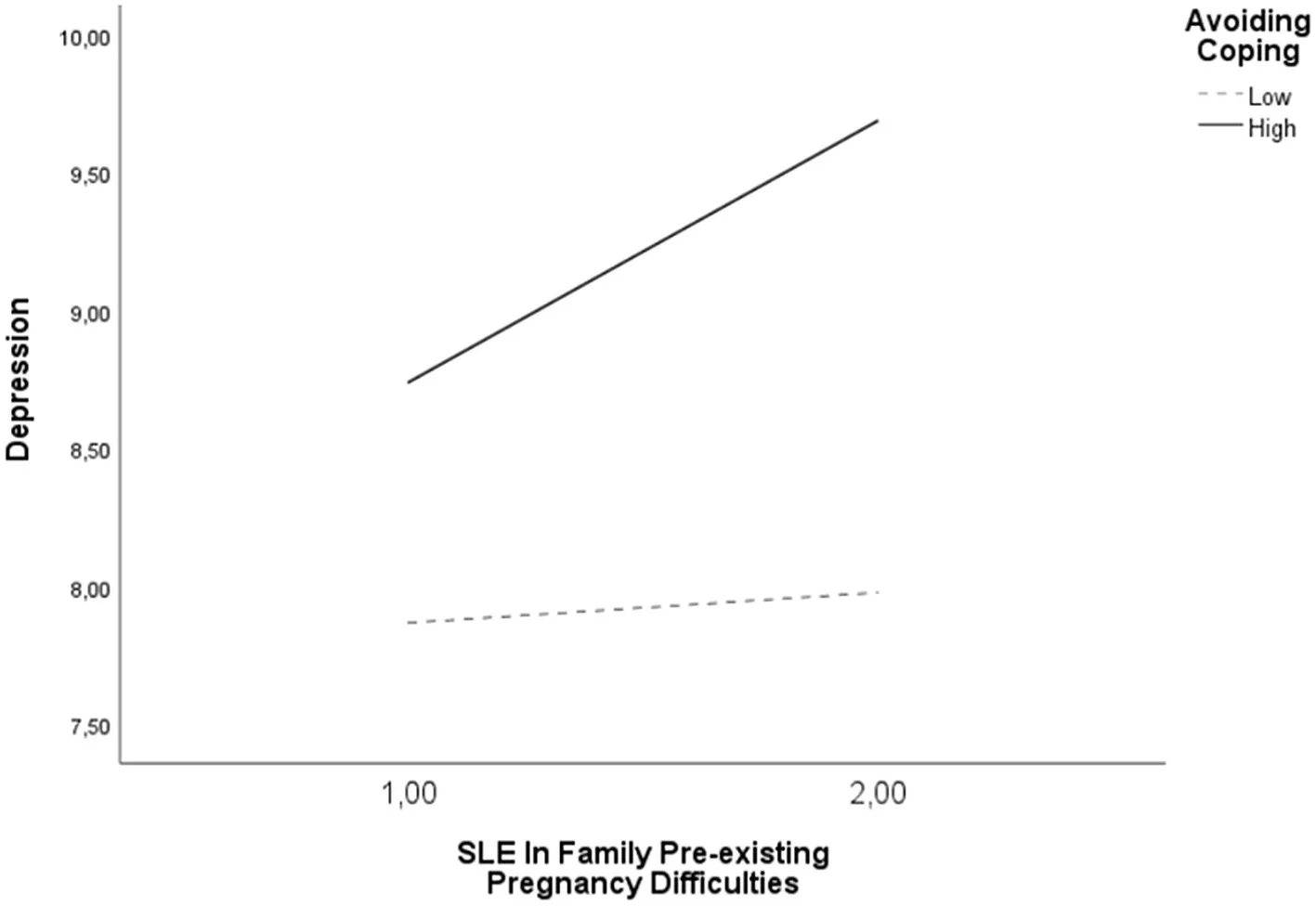
The moderating role of Avoiding Coping in the associations between SLE-in family pre-existing. Pregnancy difficulties and depression. Avoiding coping is displayed at low (−1 SD) and high (+1 SD) levels.

**Figure 6:**
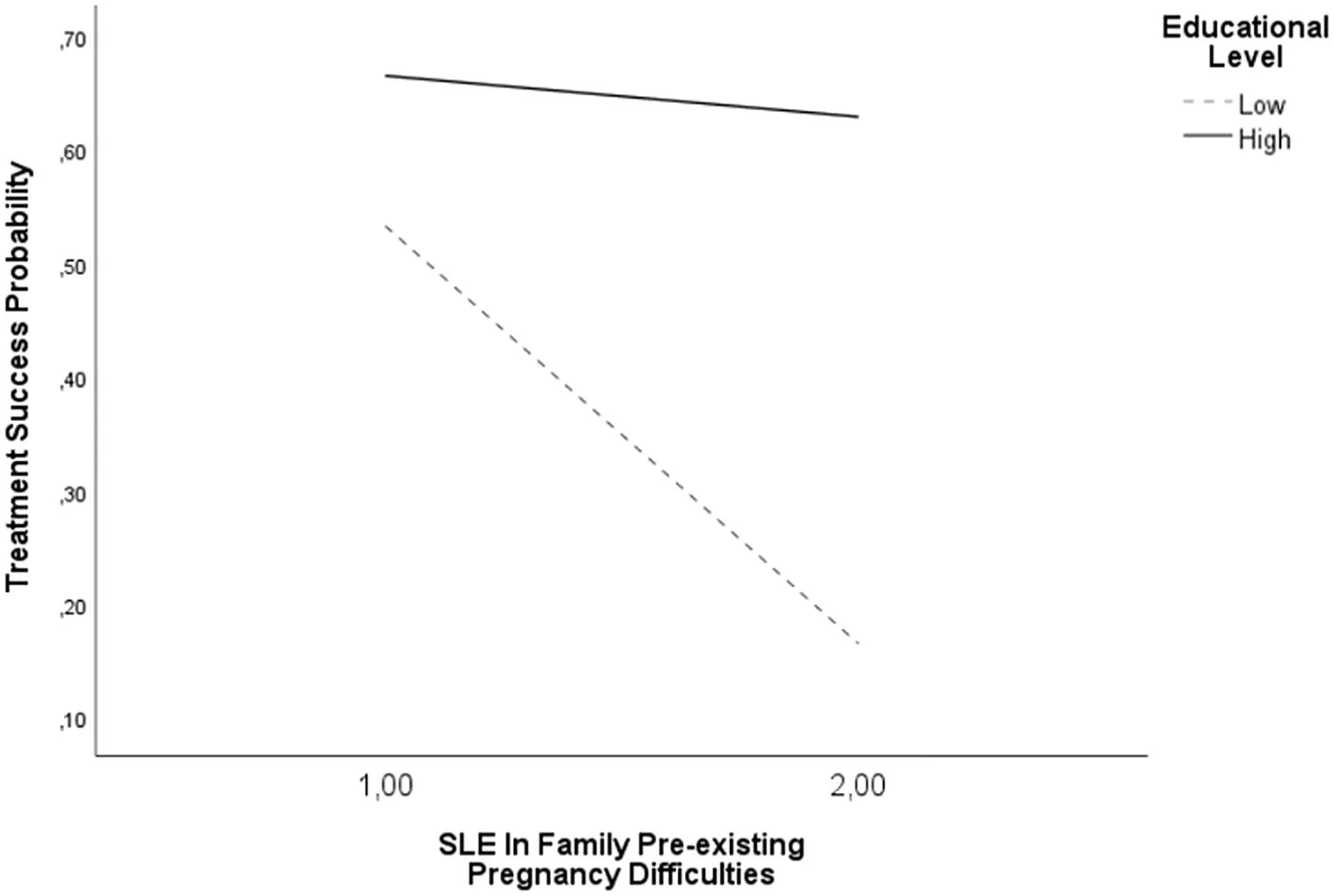
The moderating role of educational level in the associations between SLE-in family pre-existing p. Pregnancy difficulties and treatment outcome. Educational level is displayed at low (−1 SD) and high (+1 SD) levels.

With respect to H3, the data highlighted specific moderating effects which varied among the different models including each of the three Stressful Life Events explored. Specifically, considering *In Family Pre-existing Pregnancy Difficulties* (Figure 1) data revealed the statistically significant moderating effect of Avoiding coping in exacerbating its associations with Depression (*In Family Pre-existing Pregnancy Difficulties* × *Avoiding Coping*: Effect = 0.14, C.I. = 0.02 to 0.26; *t* = 2.42; Δ*R*^2^ = 0.04, *p* = 0.017; Figure 5). However, the data also revealed the statistically significant moderating effect of Educational Level in buffering the negative associations between *In Family Pre-existing Pregnancy Difficulties* and Treatment Success (*In Family Pre-existing Pregnancy Difficulties* × *Educational Level:* Effect = 0.31, C.I. = 0.00 to 0.63; Z = 1.98; *χ*^2^ = 4.536, *p* = 0.033; Figure 6).

**Figure 7:**
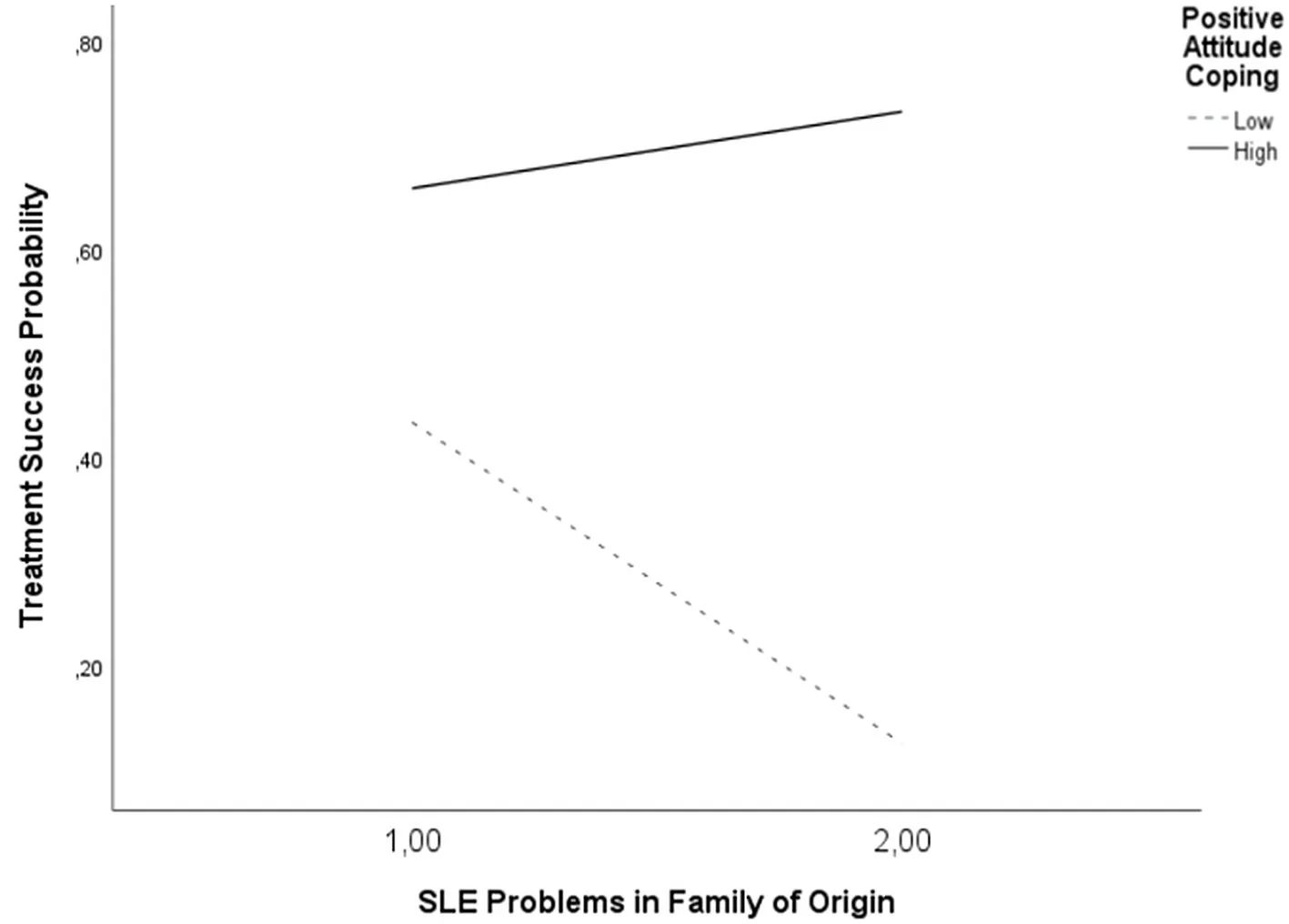
The moderating role of positive attitude coping in the associations between SLE problems in family of origin and treatment outcome. Positive attitude coping is displayed at low (−1 SD) and high (+1 SD) levels.

Considering *Problems in the Family of Origin* (Figure 2), findings revealed the statistically significant moderating effect of Positive Attitude coping strategies in buffering its negative association with Treatment Success (*Problems in the Family of Origin* × *Positive Attitude* coping: Effect = 0.25, C.I. = 0.00 to 0.50; Z = 1.93; *χ*^2^ = 4.014, *p* = 0.045; Figure 7).

**Figure 8:**
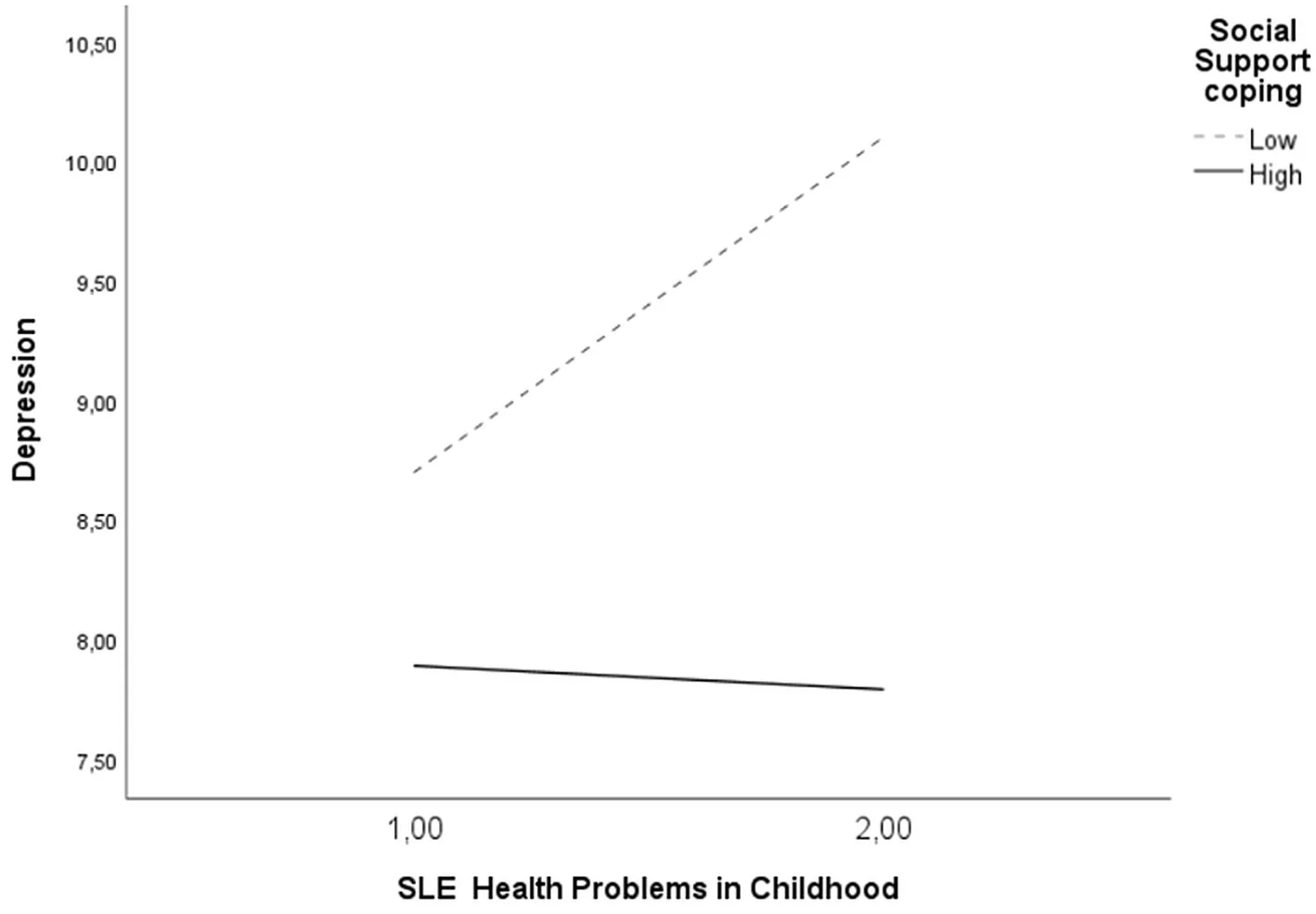
The moderating role of social support coping in the associations between SLE health problems in childhood and depression. Social support coping is displayed at low (−1 SD) and high (+1 SD) levels.

Finally, considering *Health Problems in Childhood* (Figure 3) data revealed the statistically significant moderating effect of Social Support coping in buffering its associations with Depression (*Health Problems in Childhood* × *Social Support coping:* Effect = −0.21, C.I. = − 0.35 to −0.07; *t* = 2.98; Δ*R*^2^ = 0.05, *p* = 0.003; Figure 8).

## 4 Discussion

This prospective study aimed to respond to the growing need to tailor reproductive care practices based on a multidisciplinary and evidence-based approach (Cesta et al., 2018; Cimadomo et al., 2024; Sunyach et al., 2025; World Health Organization, 2025; Zurlo et al., 2020a; Zurlo et al., 2023), thereby providing customized, team-based strategies that can help to effectively enhance reproductive health.

Firstly, with respect to H1 (i.e., main effects of SLEs on ART treatment outcomes), findings provided evidence for the associations of only one out of the three events explored with Treatment Outcome, namely *In Family Pre-existing Pregnancy Difficulties* (i.e., pregnancy or childbirth-related difficulties, such as a history of infertility, unfulfilled desire for motherhood, unplanned pregnancies, voluntary abortions, miscarriages, stillbirths, premature births and complications during pregnancy and childbirth, or children born with disabilities). Interestingly, the study provided evidence – for the first time – on the presence of specific fertility-related events in own and/or in patients’ family background as reported to a greater extent among women belonging to the *Treatment Failure* group than to the *Treatment Success* group, and also significantly associated with a lower likelihood to achieve a pregnancy. These findings suggest the possibility of a vicious cycle, in which challenges associated with fertility repeatedly recur – roughly like there is no way to break this repeating pattern (in one’s own history and in one’s transgenerational history).

From this perspective, findings aligned with research highlighting the need for accounting for individual and transgenerational dimensions (Wischmann et al., 2009; Geisler et al., 2020; Gleason et al., 2020; Zurlo et al., 2019) that can indeed increase people’s vulnerability to subsequent events (Zhou et al., 2026) and – in our study – can even significantly hinder the achievement of pregnancy. These first results should be carefully considered, given that without identifying factors influencing ART treatment outcomes – thus including but not limiting the assessment to medical parameters – many women could endure in struggling with repeated ART treatment failures (Hillcoat et al., 2023), with a significant cost for their psychological and physical health (Tadesse et al., 2025; Wade et al., 2015; Zurlo et al., 2018).

Nonetheless, a greater, more comprehensive, and complex understanding was achieved when findings from mediation (H2) and moderation (H3) analyses were integrated and discussed. Specifically, considering the associations between *In Family Pre-existing Pregnancy Difficulties* and Treatment Outcomes, data revealed not only statistically significant direct associations but also indirect effects on Treatment Outcome, *via* women’s Depressive symptoms. Therefore, this finding provided evidence extending research findings on the mediating role of psychological health conditions in linking stressful events and health outcomes (Lee et al., 2022; Li et al., 2024) also to the field of infertility research.

However, data also revealed a significant resource potentially counteracting this vicious circle, namely, *Educational Level*. Notwithstanding the role of this factor has been widely underlined in infertility research (e.g., Mahalingaiah et al., 2011; Moutzouroulia et al., 2025), its role in specifically moderating (buffering) the association between *In Family Pre-existing Pregnancy Difficulties* and Treatment outcome should be discussed in detail together with findings highlighting a higher educational background among women belonging to the *Treatment Success* group. This finding could indeed be analyzed by considering evidence that people with higher educational attainment are more likely to adjust better to events, also due to a greater use of mature defense mechanisms, such as rationalization and intellectualization, and to a lesser use of immature defense mechanisms (Blanco et al., 2023; Schneider, 2025). From this perspective, the use of immature defensive mechanisms was found to play a central role in the associations between exposure to multiple types of adverse childhood events and somatization (Ferrajão et al., 2025). Therefore, in our case, it seems sound to hypothesize that women with a higher educational level could better integrate their own or relatives’ fertility-related experience in the infertility treatment path, protecting themselves more effectively from the risk deriving from the repetition of traumatic experiences.

In line with this, still considering *In Family Pre-existing Pregnancy Difficulties*, data also revealed that its detrimental association with Depression can be further exacerbated by women’s recourse to Avoiding strategies. Once again, this evidence should be carefully considered. Indeed, research has suggested that the recourse to active avoiding/positive distraction (Kleiber et al., 2002; Waugh et al., 2020) could represent a key resource to reduce the pressure around infertility pathway effectively (Khalid and Dawood, 2020; Zurlo et al., 2020b). However, undoubtedly, in our research context, avoiding coping strategies in terms of defensive denial of one’s own/family pregnancy difficulties can only exacerbate psychological and physical suffering, raising feeling of hopelessness and sense of resignation to “one’s fate.”

Even more original findings emerged when considering the associations between the other two SLEs, namely *Problems in the Family of Origin* (e.g., maltreatment and abuses, premature deaths, financial problems, divorce) and *Health Problems in Childhood* (e.g., injuries, illness, hospitalization) with Treatment Outcome, for which no direct effects yet only “hidden” effects were observed – that is, effects entirely mediated by depression. In other words, data suggested that the experiences of these events in women’s background could significantly exacerbate the risk of depression. This psychological condition, if not addressed, could, in turn, reduce the likelihood of achieving a pregnancy. In line with this, interestingly, whereas infertility research often underlined the impact of both anxiety and depression in significantly influencing treatment experience and outcomes (Lu et al., 2025), our findings provided evidence for a central role played by depressive states. From this perspective, psychological symptoms need to be systematically and routinely assessed in medical centers by using validated measurement tools. This would allow practitioners to adequately distinguish non-clinical expressions of suffering underpinning the infertility experience from clinically relevant forms of disease. The latter could indeed play a pivotal and – sometimes – “hidden” role.

However, also for these two SLEs, and in line with previous research (Hudson et al., 2025; Zurlo et al., 2025), our findings provided evidence on specific coping resources to be accounted for when defining support interventions. Specifically, women’s recourse to Positive Attitude coping strategies was found able to buffer the detrimental influence of *Problems in the Family of Origin*, thus increasing the chance of Treatment Success. This is probably by allowing women to re-appraise and deal with infertility without this event chaining together into a series of negative past events, potentially increasing the risk of a self-fulfilling prophecy. In the same direction, data revealed the statistically significant moderating effect of Social Support coping in buffering the association between *Health Problems in Childhood* and Depression. This finding suggested that although some previous negative medical experiences may be recalled during infertility treatments, certain resources – in particular the ability to seek and find support – can prevent psychological disease escalation, potentially resulting in a higher chance of achieving a pregnancy.

Overall, these findings responded to the call for restructuring research agendas in the field of reproductive health (Hillcoat et al., 2023). It was indeed recommended to adopt a *Life-Course* perspective in reproductive research (thus including patients’ history) to unveil the interactive associations between negative experiences and adverse women’ reproductive outcomes by also exploring the complex interaction between psychological health and reproductive health (Hillcoat et al., 2023). In particular, our findings provided evidence on how *Stressful Life Events* (SLEs) and, above all, *In Family Pre-existing Pregnancy Difficulties*, can potentially have long-term influence on ART outcomes *via* women’s psychological health (Depression), along with indications for further risks (Avoiding coping strategies) and resources (Educational Level, Positive Attitude and Social Support coping strategies) to be accounted. Addressing these factors through *routine* psychological screening and multidisciplinary counselling interventions in ART centers may enhance both psychological health and treatment success for infertile women.

### 4.1 Limitations

Despite its strengths, the study has several limitations that should be addressed. Firstly, the sample consisted of Italian women belonging to heterosexual couples, thus limiting the generalizability of our findings. Considering the need to ensure the representation of as diverse a range of families as possible (Dempsey and Critchley, 2010) as well as to account for people’s cultural context (e.g., individuals from diverse backgrounds manage unpleasant emotions differently; Tamir et al., 2024), future research could be developed with a cross-cultural design and with a more heterogeneous sample of patients to test the generalizability of our results. Secondly, the data were collected by only one source (self-report measurement tools), increasing the risk of social desirability bias. In addition, the inclusion of retrospective data, namely the recall of SLEs – particularly of previous childhood events – may have provided less accurate data. Nevertheless, considering the assessment of the presence/absence of these events (rather than the different types or perceived levels of stress linked to them – that could be biased to a greater extent due to the longer retention interval), we believe that using the recall method might have affected the accuracy of our findings to a little extent. On the other side, precisely the gathering of information on SLEs only in terms of Presence/Absence, rather than by using other validated adversity instruments, have limited the possibility of deepening the types and nuances of their experience. Therefore, future research could also be designed to include data on a wider range of tools and sources of data (e.g., interviews). Moreover, considering the study design, multiple testing across analyses conducted by using Hayes’s PROCESS tool may have increased the risk of family-wise error. However, each mediation and moderation model was run separately to test distinct *a priori* hypotheses, so mitigating this risk. In addition, despite this limitation, the use of bootstrapping (5,000 resamples) ensured the accuracy of the parameters estimated within each model. Notwithstanding, still considering the study design, despite the prospective design, which allows for suggesting cause-and-effect relationships, inferences concerning the temporal associations between SLEs and ART Treatment Success should be made with extreme caution. This is also due to the presence of further potential confounding/intervening factors that were not assessed and addressed in the present study yet have a significant impact on treatment outcome. Specifically, in the present study, data on the ART treatment outcomes was collected in terms of Failure or Success and linked to psychological variables, while major reproductive variables, such as the ovarian reserve (e.g., Anti-Müllerian Hormone [AMH] and Antral Follicle Count [AFC]), patients’ health status (e.g., Body Mass Index [BMI] and health-adverse behaviors such as smoking), laboratory and embryology-related variables (e.g., embryo quality, euploid status, fertilization method), and treatment/protocol variables (e.g., type of ART treatment, stimulation protocol, fresh vs. frozen transfer, handling of multiple cycles) were not assessed and, therefore, were not adjusted for. In the same direction, at T2, patients’ information in terms of having achieved a live birth (no/yes) was collected, without gathering other relevant medical information and parameters and without assessing their psychological health conditions (Anxiety/Depression). Therefore, future research and projects should be designed and implemented by a multidisciplinary team of researchers and practitioners, so simultaneously exploring the influence of several factors – from the medical and psychological fields – potentially associated with ART treatment outcome and to patients’ reproductive health.

### 4.2 Conclusion

Psychological factors have a significant impact on key fertility-related parameters, on how women adjust to the experience of infertility, and on ART treatment outcomes. This is now increasingly supported by evidence. Yet, expanding the scope of the investigation into the impact of psychological factors to a broader timeframe – to include the Stressful Life Events (SLEs) experienced by women during their childhood and family life – seems now to be a pivotal step. This can, indeed, help researchers and practitioners gain a clearer understanding of how the current infertility experience fits into the patients’ history, in which past events may serve as the lens through which they experience and adjust to their current condition. However, the present study goes even beyond this objective. Indeed, rather than providing static and linear associations between the study variables, this work adopted a transactional approach, thus achieving a more complex understanding of patients’ authentic experience, in which multiple factors interplay to influence an outcome. Strengthened by a prospective design, this study indeed unveiled the direct but - most importantly - hidden associations between stressful life events and ART treatment outcomes (Failure/Success) as mediated by depressive states. Furthermore, the moderating evidence suggesting further risks (Avoiding Coping) yet also key resources (Educational Level, Positive Attitude and Social Support Coping) also enriched the implications for the development of tailored interventions in the context of reproductive health. In conclusion, the present study proposes to also consider the need to address the impact of SLEs on the psychological development and functioning among the significant parameters to be assessed by healthcare professionals in counselling interventions with infertile patients, to improve the psychological adjustment to treatment paths and the possibilities of treatment success.

## Data Availability

The datasets will be provided by the corresponding author upon reasonable request. Requests to access these datasets should be directed to Maria Clelia Zurlo zurlo@unina.it.

## Supplementary material

SUPPLEMENTARY FIGURE S1 ROC curves of the regression models predicting Treatment Outcomes: **(a)** SLE-pre-existing pregnancy difficulties and depression; **(b)** SLE-problems in family of origin and depression; **(c)** SLE-health problems in childhood and depression. The red lines illustrate the predictive models (AUC > 0.70 for all predictive models).
