# Supplementary material for: Effects of stressful life events on ART treatment outcomes: a prospective study on the role of individual and personality characteristics and psychological health conditions
Source: Front Psychol. 2026 Aug 31;17:1915014. doi: 10.3389/fpsyg.2026.1915014 (PMC13572596; doi:10.3389/fpsyg.2026.1915014)
Supplement: Supplementary file 1 [file Table_1.docx]

**Supplementary Table 1.** Mediating analyses: path coefficients

| Independent  Variable  (X) | Mediator  (M) | Dependent Variable  (Y) | Path A | | | | | Path B | | | | Direct Effect | | | | | Indirect Effect | |
| --- | --- | --- | --- | --- | --- | --- | --- | --- | --- | --- | --- | --- | --- | --- | --- | --- | --- | --- |
|  |  |  | Coff. (se) | *t* | *p* | [95% C.I.] | Coff. (se) | | *Z* | *p* | [95% C.I.] | Coff. (se) | *Z* | *p* | [95% C.I.] | Coff. (se) | | [95% C.I.] |
| In Family Pre-existing Pregnancy Difficulties | Depression | Treatment Success | 0.73 (0.26) | 2.80 | 0.005 | [0.21, 1.25] | -0.78 (0.17) | | -4.42 | 0.000 | [-1.12, -0.43] | -0.94 (0.45) | -2.10 | 0.035 | [-1.82, -0.06] | -0.57 (0.27) | | [-1.19, -0.13] ^a^ |
|  |  |  | **Model Summary** | | | | **-2LL** | | **ModelLL** | **df** | ***p*** | **McFadden** | **CoxSnell** | **Nagelkrk** |  |  | |  |
|  |  |  |  |  |  |  | 144.084 | | 35.641 | 2.000 | 0.000 | 0.198 | 0.239 | 0.320 |  |  | |  |
| Problems in Family of Origin | Depression | Treatment Success | 0.66 (.24) | 2.68 | 0.008 | [0.17, 1.15] | -0.79 (0.17) | | -4.53 | 0.000 | [-1.14, -0.45] | -0.23 (0.40) | -0.56 | 0.572 | [-1.02, 0.56] | -0.53 (0.23) | | [-1.07, -0.13] ^b^ |
|  |  |  | **Model Summary** | | | | **-2LL** | | **ModelLL** | **df** | ***p*** | **McFadden** | **CoxSnell** | **Nagelkrk** |  |  | |  |
|  |  |  |  |  |  |  | 148.321 | | 31.404 | 2.000 | 0.000 | 0.174 | 0.214 | 0.286 |  |  | |  |
| Health Problems in Childhood | Depression | Treatment Success | 0.60 (.25) | 2.40 | 0.017 | [0.10, 1.10] | -0.81 (0.17) | | -4.61 | 0.000 | [-1.15, -0.46] | -0.10 (0.41) | -0.26 | 0.793 | [-0.91, 0.70] | -0.49 (0.23) | | [-1.03, -0.09] ^b^ |
|  |  |  | **Model Summary** | | | | **-2LL** | | **ModelLL** | **df** | ***p*** | **McFadden** | **CoxSnell** | **Nagelkrk** |  |  | |  |
|  |  |  |  |  |  |  | 148.571 | | 31.154 | 2.000 | 0.000 | 0.173 | 0.213 | 0.284 |  |  | |  |

*Notes.* Path A = Effect of independent variable on mediator; Path B = Effect of mediator on dependent variable; Direct effect = effect of independent variable on dependent variable controlling for the mediator; Indirect effect = Effect of independent variable on dependent variable through the mediator. ^a^ Partial Mediation, ^b^Full mediation. Only significant mediation models are displayed.
